# Whole-genome sequence analysis and comparisons between drug-resistance mutations and minimum inhibitory concentrations of *Mycobacterium tuberculosis* isolates causing M/XDR-TB

**DOI:** 10.1371/journal.pone.0244829

**Published:** 2020-12-31

**Authors:** Ditthawat Nonghanphithak, Orawee Kaewprasert, Pratchakan Chaiyachat, Wipa Reechaipichitkul, Angkana Chaiprasert, Kiatichai Faksri

**Affiliations:** 1 Department of Microbiology, Faculty of Medicine, Khon Kaen University, Khon Kaen, Thailand; 2 Research and Diagnostic Center for Emerging Infectious Diseases (RCEID), Khon Kaen University, Khon Kaen, Thailand; 3 Department of Medicine, Faculty of Medicine, Khon Kaen University, Khon Kaen, Thailand; 4 Drug Resistant Tuberculosis Research Fund Laboratory, Research and Development Affairs, Faculty of Medicine, Siriraj Hospital, Mahidol University, Bangkok, Thailand; St Petersburg Pasteur Institute, RUSSIAN FEDERATION

## Abstract

Drug resistance (DR) remains a major challenge for tuberculosis (TB) control. Whole-genome sequencing (WGS) provides the highest genetic resolution for genotypic drug-susceptibility tests (DST). We compared DST profiles of 60 *Mycobacterium tuberculosis* isolates which were drug resistant according to agar proportion tests (one poly DR-TB, 34 multidrug-resistant TB and 25 extensively drug-resistant TB). We additionally performed minimum inhibitory concentration (MIC) tests using Sensititre MYCOTBI plates (MYCOTB) and a WGS-based DST. Agreement between WGS-based DST and MYCOTB was high for all drugs except ethambutol (65%) and ethionamide (62%). Isolates harboring the -15 c/t *inhA* promoter mutation had a significantly lower MIC for isoniazid than did isolates with the *katG* Ser315Thr mutation (p < 0.001). Similar patterns were seen for ethambutol (*embB* Gly406Asp vs. *embB* Met306Ile), streptomycin (*gid* Gly73Ala vs. *rpsL* Lys43Arg), moxifloxacin (*gyrA* Ala90Val vs. *gyrA* Asp94Gly) and rifabutin (*rpoB* Asp435Phe/Tyr/Val vs. *rpoB* Ser450Leu). For genotypic heteroresistance, isolates with lower proportion of mapped read tended to has lower MIC of anti-TB drugs than those with higher proportion. These results emphasize the high applicability of WGS for determination of DR-TB and the association of particular mutations with MIC levels.

## Introduction

Emergence of drug-resistant (DR) strains of *Mycobacterium tuberculosis* (*Mtb*) remains the challenge for tuberculosis (TB) control. In 2018, the World Health Organization (WHO) estimated that there were 457,000 multidrug-resistant TB (MDR-TB) cases globally and that 8.5% of these were extensively drug-resistant TB (XDR-TB) [1]. Early identification of TB and accurate drug-susceptibility testing (DST) are urgently required for appropriate TB treatment and to reduce the risk of further DR-TB development.

The gold standard of DST for *Mtb* is the proportional method [2]. The minimum inhibitory concentration (MIC) test is another phenotypic method for quantification of the resistance level. Such phenotypic DSTs are time-consuming and laborious. Hence an alternative approach, genotypic DST, is becoming more readily accepted, provided that the complete database of mutations associated with drug resistance is available. Whole-genome sequencing (WGS) provides the best resolution of the genetic repertoire and is highly applicable for predicting drug-resistance profiles of *Mtb* and simultaneously can determine clustering for transmission analysis [3, 4]. There have been few direct comparisons of these three DST methods [5], especially for second-line drugs.

Quantitative phenotypic resistance (indicated by MIC values) associated with different mutations has been reported [5–7]. The current guidelines from WHO suggest that mutations detected in *Mtb* isolates can be used to predict resistance levels [5]. However, knowledge of such mutations is still limited in both number of tested strains and number of drugs available in the WHO database, and again especially for the second-line drugs [8].

Heteroresistance of *Mtb*, the mixture of susceptible and resistant strains in a single sample [9], has an effect on quantitative DSTs [10, 11]. A previous study compared different phenotypic DSTs to detect heteroresistance to rifampicin (RIF) [10] and genotypic approaches using WGS have also been described [11]. However, the few relevant studies have not made direct comparisons between genotypic heteroresistance (based on variant frequencies) and MIC levels for *Mtb*.

Thus, we compared DST profiles of a collection of M/XDR-TB *Mtb* isolates from Thailand, using phenotypic methods (agar proportion and MIC tests using MYCOTB) and a genotypic method (WGS analysis). The association between specific mutations and levels of drug resistance was analyzed for 11 drugs, including isoniazid (INH), RIF, ethambutol (EMB), streptomycin (STR), second-line injectable drugs (SLIDs): kanamycin (KAN) and amikacin (AMK), fluoroquinolones: ofloxacin (OFX) and moxifloxacin (MXF), ethionamide (ETO), *para*-aminosalicylic acid (PAS) and rifabutin (RFB). The possibility of genotypic heteroresistance, based on variant frequencies and quantitative MIC levels, was also investigated.

## Materials and methods

### *Mtb* isolates and setting

Sixty clinical *Mtb* isolates collected between 2003 and 2017 were obtained from stock cultures deposited at the Drug-Resistant Tuberculosis Research Fund, Faculty of Medicine, Siriraj Hospital, Mahidol University, Bangkok, Thailand. The clinical specimens were stained for acid-fast bacilli using the Kinyoun method and subjected to phenotypic DST using the agar proportion method. Each selected isolate was from a different patient and each isolate was resistant at least to RIF (Poly-DR TB, n = 1; MDR, n = 28; Pre-XDR, n = 6 and XDR, n = 25). All isolates were sub-cultured on Löwenstein–Jensen media and incubated at 37°C for four to six weeks. Multiple loops of mycobacterial culture were used for genomic DNA extraction (using the cetyl-trimethyl-ammonium bromide-sodium chloride method [12]) and for MIC-based phenotypic DSTs. This study was approved by the Khon Kaen University Ethics Committee in Human Research (Ethics number HE601249).

### Phenotypic drug susceptibility testing

The standard agar proportion method was performed according to recommendations from the US Centers for Disease Control and Prevention [13]. Briefly, anti-TB drug discs were placed into the centers of individual quadrants of sterile plates, then 5.0 ml of Middlebrook 7H10 (Difco, Detroit, MI, USA) containing 10% oleic acid-albumin dextrose-catalase (BBL, Becton Dickinson, USA) was poured over the plate, and the agar was allowed to solidify overnight at room temperature. The inoculum was prepared by suspending the *Mtb* colonies in Middlebrook 7H9 (Difco, Detroit, MI, USA) and adjusting the supernatant to turbidity equivalent to a MacFarland standard of one. The suspension was diluted to 10^−2^ and 10^−4^ [13]. The dilutions were inoculated onto the control quadrant, drug-free medium, and drug-containing quadrants. The plate was incubated at 37°C until colonies appeared on the control quadrant after approximately two to four weeks. Percentage of resistance was determined by (no. of colonies on drug-containing quadrant/no. of colonies on control quadrant)×100. An isolate was regarded as resistant when the percentage of resistance was ≥1%.

The MIC-based phenotypic DST was performed using Sensititre MYCOTBI (MYCOTB) plates according to the manufacturer (TREK Diagnostic Systems, West Sussex, United Kingdom). The wells of a MYCOTB plate contain 12 lyophilized anti-TB drugs with ranges of drug concentrations appropriate to each drug [14, 15]. Briefly, *Mtb* colonies were suspended in saline-Tween with glass beads for agitation and the turbidity of the supernatant adjusted to 0.5 MacFarland standard. This suspension (100 μl) was added into Middlebrook 7H9 medium and 100 μl of this mixture was added into each well of the MYCOTB plate. The plates were covered with plastic seals and incubated at 37°C. The plates were read using the Sensititre Vizion Digital MIC Viewing System (TREK Diagnostic Systems) at 10 days, or 21 days if poor growth was observed. The MIC was defined as the lowest concentration of anti-TB that inhibits visible growth.

The critical concentrations (CCs) used for agar proportion and MYCOTB assays are listed in S1 Table. All isolates were tested once: If the test failed, it was repeated. *Mtb* H37Rv ATCC 27294 strain was used as a control for both agar proportion and MYCOTB assays.

### Whole-genome sequencing and *in silico* detection of drug resistance

WGS was done for a subset (n = 27) of the 60 genomic DNA samples at the Genome Institute of Singapore, Singapore, using the TrueSeq DNA sample preparation kit (Illumina, San Diego, CA) and the MiSeq platform (Illumina) generating 250-bp paired-end reads, or using the NEBnext Ultra kit (Illumina, San Diego, CA) for the HiSeq (Illumina) platform generating 150-bp paired-end reads. The remaining 33 samples were sequenced at NovogeneAIT, N.T., Hong Kong, using the HiSeq (Illumina) platform generating 150-bp paired-end reads. The quality of sequence reads was determined using FastQC version 0.11.7 [16]. The sequencing coverage and percentage of mapped reads against the reference genome of the H37Rv strain were determined using GATK version 3.4.0 [17] and SAMtools version 0.1.19 [18]. The mean genome coverage and the mean mapping rate were 224.5 (±152.4 standard deviation) and 97.9%, respectively. The WGS data are available in the Sequence Read Archive (https://www.ncbi.nlm.nih.gov/sra) with the accession Nos. PRJNA598981 and PRJNA598949.

To detect drug resistance and determine *Mtb* lineage from the WGS data, raw fastq files were uploaded to an online tool, TB-Profiler version 2.8.6 [19]. To detect heteroresistant isolates, manual analysis was done to calculate frequencies of variants occurring in fewer than 100% of reads. Paired-end raw reads of each isolate were mapped to the *Mtb* H37Rv reference genome (GenBank accession number: NC_000962.3) using BWA-MEM version 0.7.12 [20]. SAMtools was used for SAM-BAM format conversion and sorting of mapped sequences. Local realignment of the mapped reads was performed using GATK. Variants, including single nucleotide polymorphisms (SNPs) and small indels, were called using GATK and SAMtool tools. Variant sites were filtered based on the following criteria: mapping quality >50 (-C in Samtools calling), base quality/base alignment quality >20 (-Q in Samtools calling), >10 reads or ≤2,000 reads (-d in Samtools filter) covering each site. To maximize specificity, the called variants were selected from the intersection of those identified by Samtools and GATK. For detection of heteroresistance, an in-house python script was used to extract the read frequencies supporting the mutations from the mapped reads. When read frequencies of mutant alleles were less than 99% compared to the wild-type background, we regarded this as WGS-based evidence of heteroresistance in that isolate [11]. In addition, the online tool, PhyresSE version 1.0 [21], was used for validation of drug resistance-conferring mutations obtained from TB-Profiler and for detection of heteroresistant TB.

Phylogenetic analysis of the 7,880 high-confidence SNPs identified among the 60 *Mtb* isolates was performed based on the maximum likelihood method with a general time-reversible and gamma distribution model (selected model based on data) using MEGA version 10.1 [22]. The phylogenetic tree was constructed based on 1,000 bootstrap replicates. The visualization of the phylogenetic tree was performed using iTOL [23].

### Data analysis

For all analyzes and visualization, R version 3.6.1 was used and p-values <0.05 were considered statistically significant. Sensitivity, specificity and categorical agreement with 95% confidence intervals (95% CI) were analyzed using the package epiR version 1.0–4. CompareTests version 1.2 was used for comparisons between DST methods for each drug. Analyses for INH and RIF were not performed because few or no susceptible isolates were available. Also, analyses for RFB, pyrazinamide (PZA) and D-cycloserine were not done due to lack of DST results for these from the agar proportion assay. Any association between MIC data and the drug resistance-conferring mutations was tested using the Wilcoxon rank-sum test. Graphs representing genetic information and their corresponding MICs were plotted using package ggplot2 version 3.2.1.

## Results

### Characteristics of the studied isolates

The clinical *Mtb* isolates used were isolated from 60 TB patients. Most of the patients were male (79%). The average age was 43.6 years. Based on phylogenetic analysis, 88.3% (n = 53) of the isolates belonged to lineage 2 (East-Asian). There were two small clusters, each of two genetically identical isolates: only in one of these did the isolates share the same drug-resistance patterns (S1 Fig).

### Agreement of DST results between phenotypic and genotypic methods

Agreement, sensitivity and specificity among DST methods are shown (Table 1). High levels of agreement between the agar proportion and WGS-based DSTs were found for OFX (95%) and AMK (90%) (Table 1). Agreement between WGS-based DST and MYCOTB was high for all drugs except EMB (65%) and ETO (62%).

**Table 1:** Agreement among phenotypic and genotypic DST assays.

Drug	WGS	Agar prop.		WGS vs. Agar prop.			WGS	MYCOTB		WGS vs. MYCOTB		
				% Sensitivity (95% CI)	% Specificity (95% CI)	% Categorical agreement (95% CI)				% Sensitivity (95% CI)	% Specificity (95% CI)	% Categorical agreement (95% CI)
		R	S					R	S			
Isoniazid^a^	**R**	56	0	NA	NA	NA	**R**	54	2	NA	NA	NA
	**S**	3	1				**S**	2	2			
Rifampicin^a^	**R**	57	0	NA	NA	NA	**R**	51	6	NA	NA	NA
	**S**	3	0				**S**	0	3			
Ethambutol^b^	**R**	35	4	92 (78–97)	79 (55–92)	88 (77–94)	**R**	21	21	100 (NA)	46 (31–62)	65 (54–75)
	**S**	3	15				**S**	0	18			
Streptomycin	**R**	34	9	94 (80–99)	63 (42–79)	82 (71–89)	**R**	39	4	98 (84–100)	80 (57–92)	92 (82–96)
	**S**	2	15				**S**	1	16			
Kanamycin^b^	**R**	19	0	70 (50–85)	100 (NA)	86 (76–93)	**R**	19	0	95 (62–100)	100 (NA)	98 (79–100)
	**S**	8	32				**S**	1	40			
Amikacin	**R**	17	0	74 (52–88)	100 (NA)	90 (80–95)	**R**	17	0	94 (60–99)	100 (NA)	98 (79–100)
	**S**	6	37				**S**	1	42			
Ofloxacin	**R**	28	0	90 (72–97)	100 (NA)	95 (84–99)	**R**	28	0	97 (71–100)	100 (NA)	98 (79–100)
	**S**	3	29				**S**	1	31			
Moxifloxacin^b^	**R**	15	11	88 (63–97)	73 (58–84)	78 (65–86)	**R**	24	4	96 (76–99)	89 (73–96)	92 (82–96)
	**S**	2	30				**S**	1	31			
Ethionamide	**R**	23	6	92 (73–98)	83 (67–92)	87 (76–93)	**R**	6	23	100 (NA)	57 (44–70)	62 (49–73)
	**S**	2	29				**S**	0	31			
PAS	**R**	22	1	71 (53–84)	97 (79–100)	83 (73–90)	**R**	20	3	80 (60–91)	91 (77–97)	87 (76–93)
	**S**	9	28				**S**	5	32			

S, susceptible; R, resistant; Agar prop., agar proportion method; NA, not applicable; PAS, *para*-aminosalicylic acid.

^a^ The number of sensitive isolates based on agar proportion and MYCOTB (MIC-based DST) assays was too low (<10 isolates) to allow for reliable estimation of agreement, sensitivity and specificity.

^b^ DST results were available for all 60 isolates, except that results for ethambutol, kanamycin and moxifloxacin using agar proportion were only available for 57, 59 and 58 isolates respectively.

### Comparison between WGS-based genotypic DST and MIC results for each drug

#### Rifampicin (RIF) and rifabutin (RFB)

**Fig 1:**
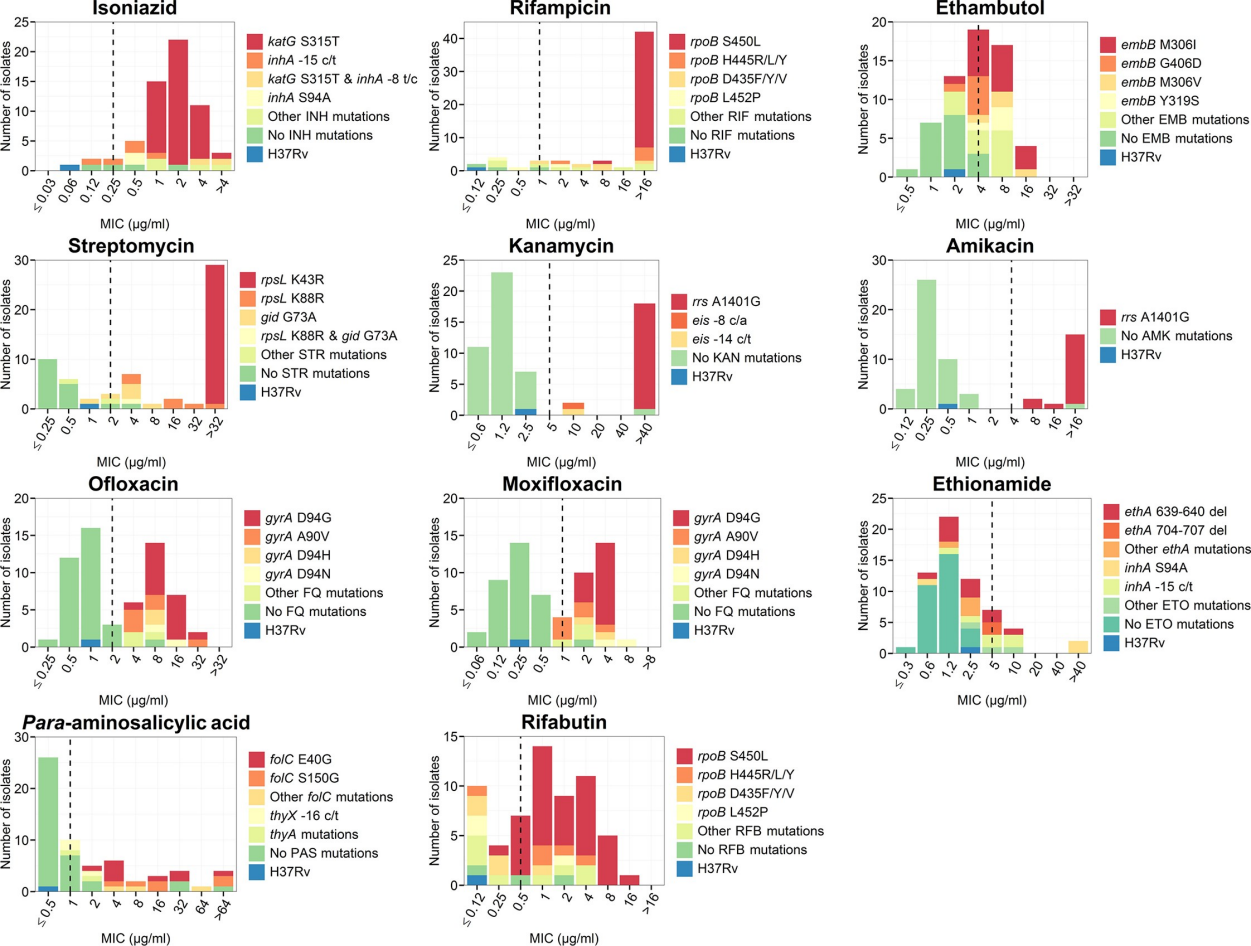
Distributions of drug resistance-conferring mutations with corresponding MIC values. Each stacked column represents a collection of isolates colored by different genetic background. The dashed lines indicate the critical concentrations used for MYCOTB. The H37Rv control strain was susceptible to all anti-tuberculosis drugs and represents the wild-type.

The *rpoB* Ser450Leu mutation was commonly found (n = 36, 60%) among both RIF- and RFB-resistant isolates (Fig 1 and S2 Table). However, only RIF-resistant isolates showed distinct MIC values beyond the CC. Many RFB-resistant isolates (n = 18) with *rpoB* mutations (e.g. *rpoB* Asp435Val, Ser441Leu, Leu452Pro) had MIC values below the CC. Isolates with *rpoB* Ser450Leu and Asp435Phe exhibited RIF resistance but were RFB-susceptible according to the MIC test.

**Fig 2:**
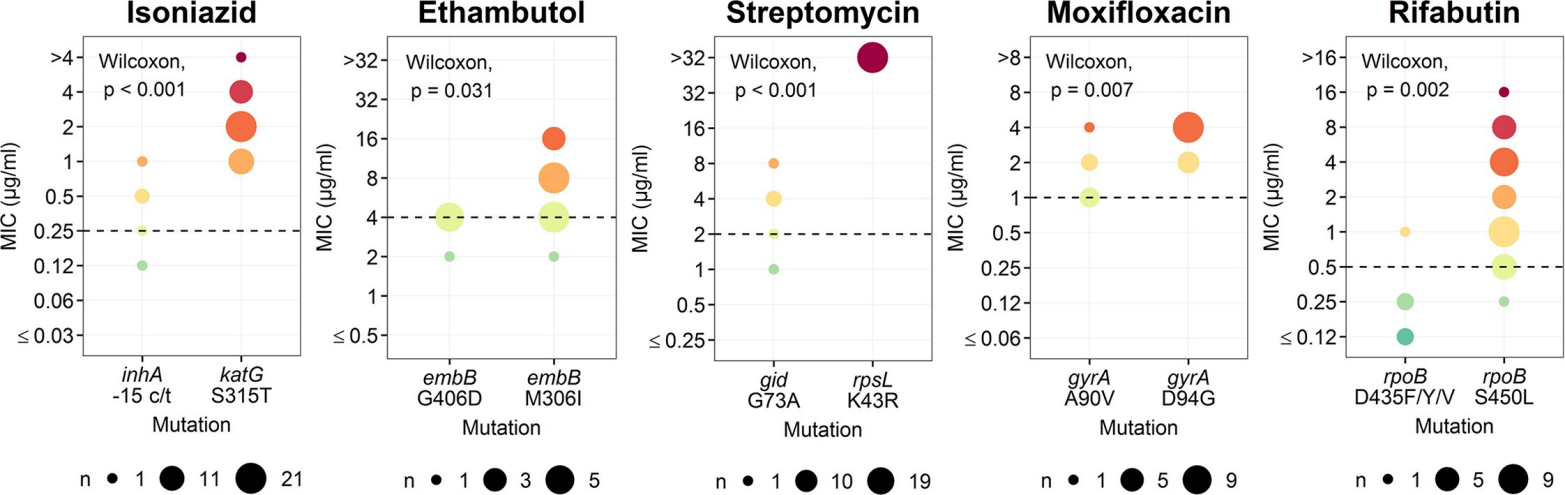
Comparisons between resistance-conferring mutations and MIC values of anti-TB drugs. Only those anti-TB drugs are shown for which common mutations are associated with significant differences in MIC levels. The dashed lines indicate the critical concentrations used for MYCOTB. The size of each circle is proportional to the number of isolates. The color of circles indicate the MIC level from low (blue-green) to high (red).

**Fig 3:**
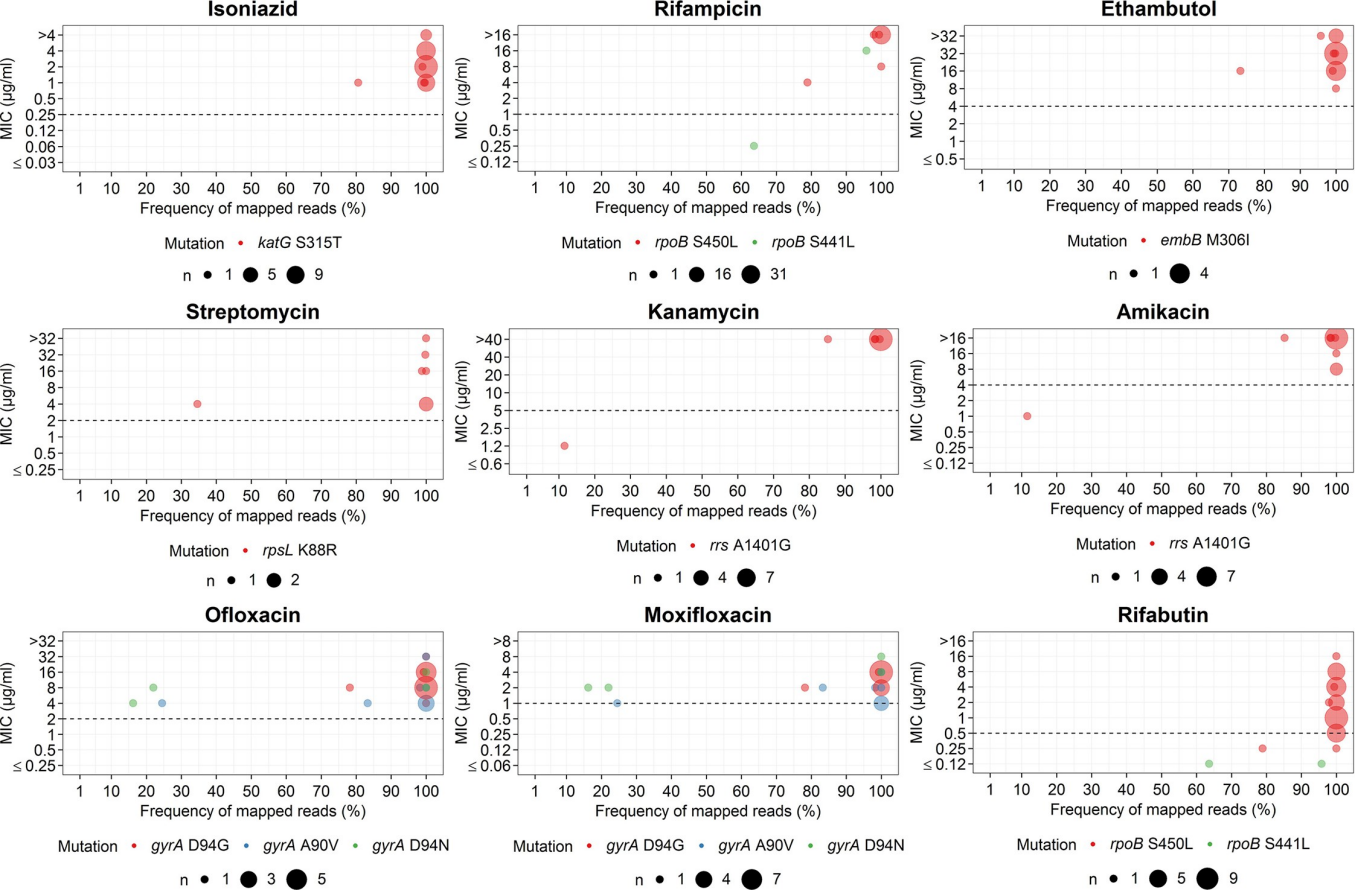
Comparison between heteroresistance (inferred from read frequencies of relevant SNPs) and MIC levels of *Mtb*. Comparisons between heteroresistance (inferred from read frequencies of relevant SNPs) and MIC levels for each anti-TB drug. The dashed line indicates the critical concentrations used for MYCOTB. Only anti-TB drugs against which heteroresistance was inferred based on read frequencies are shown. The size of each circle is proportional to the number of isolates.

Mutations in *rpoB* Asp435Phe/Tyr/Val had MIC values for RFB significantly lower than isolates with *rpoB* Ser450Leu (0.12–1 μg/ml vs. 0.25–16 μg/ml, p = 0.002) (Fig 2). One heteroresistant isolate (79% of reads support *rpoB* Ser450Leu) had MIC of RIF lower than other isolates but had a MIC value below the CC of RFB (Fig 3 and S2 Table). An isolate with 64% reads of Ser441Leu was susceptible to RIF, whereas another isolate with the same mutation (in 96% of reads) was resistant to RIF (Fig 3). However, these two isolates were both susceptible to RFB.

#### Isoniazid (INH) and ethionamide (ETO)

The most frequent mutation for INH resistance was *katG* Ser315Thr (n = 43, 72%) (Fig 1 and S2 Table). Most isolates with known INH mutations exhibited a MIC values above the CC, except for those harboring *inhA* promoter nutation alone. Two isolates without known INH resistance had MICs higher than the CC. Isolates with the -15 c/t *inhA* promoter mutation had MIC values for INH significantly lower than isolates with *katG* Ser315Thr (0.12–1 μg/ml vs. 1->4 μg/ml, p < 0.001) (Fig 2). In addition, one INH-resistant isolate with 81% read frequency of the *katG* Ser315Thr mutation had an MIC value (1 μg/ml) lower than those with 99–100% reads of this mutation (range = 1–2 μg/ml) (Fig 3).

Most (23/29, 79%) isolates with known resistance mutations for ETO (*ethA* and *inhA* promoter) had MIC values lower than the CC (Fig 1 and S2 Table). Six isolates (21%) with known ETO-resistance mutations had MIC values above the CC and five of them had resistant DST results for both the agar proportion and the MIC tests.

#### Ethambutol (EMB) and streptomycin (STR)

Half of the isolates with EMB-resistance mutations (19 in *embB* and 2 in *embA*) had MIC values below the CC (Fig 1 and S2 Table). The agreement between WGS-based DST and MIC was increased from 65% to 85% when the CC was adjusted from 4 μg/ml to 2 μg/ml. The isolates with *embB* Gly406Asp had significantly lower MIC values for EMB compared to those with *embB* Met306Ile (2–4 μg/ml vs. 2–16 μg/ml, p = 0.031) (Fig 2). In addition, one isolate with 73% heteroresistance of *embB* Met306Ile exhibited an EMB-resistant phenotype with 16 μg/ml of MIC (Fig 3).

For STR, isolates with most common mutations (*rpsL* Lys43Arg and Lys88Arg) had MIC values above the CC (Fig 1 and S2 Table). However, half of the isolates with *gid* mutations had MIC values for STR lower than the CC. The isolates with *gid* Gly73Ala had MIC values for STR significantly lower than isolates with *rpsL* Lys43Arg (1–8 μg/ml vs. >32 μg/ml, p < 0.001) (Fig 2). One isolate with *gid* Gly73Ala (100% reads) and 35% heteroresistance of *rpsL* Lys88Arg was resistant to EMB (Fig 3).

*Kanamycin (KAN) and amikacin (AMK)*. For KAN and AMK, all isolates (n = 19 and 17 for KAN and AMK, respectively) with known mutations had MIC values above the CC (Fig 1 and S2 Table). One isolate without known mutations for any of the SLIDs carried 85% reads of *rrs* A1401G (identified by in-house analysis) and this isolate was phenotypically resistant to both KAN and AMK (Figs 1, 3 and S2 Table). In contrast, another isolate carrying 12% reads of *rrs* A1401G had MIC values (1.2 μg/ml and 1 μg/ml for KAN and AMK, respectively) lower than other phenotypically KAN- and AMK-resistant isolates with high read frequencies for this mutation (KAN: 85–100% reads with MIC >40 μg/ml; AMK: 85–100% reads with MIC 8->16 μg/ml) (Fig 3).

#### Fluoroquinolones

All isolates with known *gryA* mutations were resistant to OFX but not MXF. Six isolates with *gyrA* Ala90Val had MIC values around the CC of MXF (Fig 1) that were significantly lower than isolates with *gyrA* Asp94Gly (1–4 μg/ml vs. 2–4 μg/ml, p = 0.007) (Fig 2). Discrepancy between WGS-based DST and MIC values for MXF was diminished when the CC was adjusted from 1 μg/ml to 0.5 μg/ml (Fig 1). One isolate which was genotypically wild-type (according to web-based tools) but carrying heteroresistance detected by in-house analysis (78% and 22% reads of *gryA* Asp94Gly and Asp94Asn, respectively) was resistant to both OFX and MXF (Figs 1 and 3). In addition, genotypic heteroresistance found in *gryA* mutations (Asp94Gly, Ala90Val and Asp94Asn) increased MIC values above the CC for OFX (Fig 3). In contrast, one isolate with 25% heteroresistance and five resistant isolates with 100% reads harboring *gryA* Ala90Val had MIC values at the borderline of the CC for MXF.

#### *Para*-aminosalicylic acid (PAS)

Most of the isolates with known mutations conferring PAS resistance, especially *folC*, had MIC values higher than the CC (Fig 1 and S2 Table). However, five isolates without known resistance mutations were resistant to PAS.

## Discussion

We compared the DST patterns of M/XDR-TB isolates from Thailand using different DST methods including agar proportion tests, MYCOTB (MIC tests) and WGS analysis. Low levels of agreement among these methods were noted for some drugs, especially EMB and ETO. For EMB the agreement between WGS and MYCOTB was low (65%). Possibly the CC (4 μg/ml) used for EMB is too high [24]. When we reduced the CC of EMB to 2 μg/ml, the agreement between MYCOTB vs. WGS was greatly improved (85%). Adjustment of some CCs for MIC-based DSTs might be helpful to improve the agreement between MIC-based DSTs and other methods. For ETO, there was also poor agreement between MYCOTB and WGS methods (62%), but high agreement (87%) between agar proportion and WGS methods. Such discrepancies might be due to an inappropriate CC value for ETO and/or known resistance mutations in *ethA* and the *inhA* promoter might not be associated with ETO resistance in our cohort [25, 26]. Besides an inappropriate CC value and the potential effect of previously unknown mutations or overweighted mutations, the discrepancies between DST methods might also be caused by undetected laboratory error. Taken together, these results identify drugs for which sensitivity tests might be particularly difficult to interpret and the properties of particular DST methods that might contribute to this difficulty.

Although we used CC values close to those recommended by the WHO, genotypically resistant and genotypically susceptible *Mtb* isolates were found with MIC values either side of the CC for many drugs including EMB, ETO and RFB. For example, this applied to isolates with *embB* mutations using the CC value (4 μg/ml) suggested in the test kit instructions. When the WHO-recommended CC value (5 μg/ml) was applied, discordance between genotypic and phenotypic tests was even greater for EMB. Similarly, the agreement of EMB between phenotypic and genotypic DST was low [24]. For ETO, we found isolates that had resistance-conferring mutations in the *ethA* gene and the *inhA* promotor but had MIC values lower than the CC (5 μg/ml). Mutations in the *inhA* promotor confer only low resistance levels against INH [27], and likely also against ETO. For RFB, many isolates with *rpoB* mutations had MIC values both higher and lower than the CC. Although RIF and RFB belong to the same family of anti-TB drugs, the MIC distributions relative to CCs of isolates harboring known *rpoB* mutations were not the same for both drugs. No wild-type isolates had an MIC above the CC (1 μg/ml) for RIF and few isolates with *rpoB* mutations fell below the CC. However, in the case of RFB, a greater proportion of isolates harboring *rpoB* mutations had MIC values lower than the CC (0.5 μg/ml as recommended by the kit instructions). Possibly, mutations (especially *rpoB* Asp435Val) assumed to confer resistance to RIF might not be highly correlated with RFB resistance, as found previously by others [26, 28, 29]. Furthermore, we found that isolates carrying *rpoB* Asp435Val alone had significantly lower MIC values for RFB than did isolates carrying *rpoB* Ser450Leu. Similarly, a previous study reported that *rpoB* Asp435Val alone had lower IC_50_ values for RIF and RFB than did isolates with *rpoB* Ser450Leu [29]. For STR, eight isolates with *gid* mutations had MIC values between 0.5 and 8 μg/ml, thus falling on and either side of the CC (2 μg/ml). The *gid* mutations have been determined as moderate-confidence mutations for STR resistance [30]. Possibly, mutations in *gid* confer low resistance levels. In the case of AMK and KAN, most isolates lacking specific mutations had MIC values below the CC, whereas MICs for isolates with resistance-conferring mutations fell above the CC. In addition, one isolate with no known mutations for SLIDs (tested by both *in silico* tools) exhibited heteroresistance of *rrs* A1401G (identified by in-house analysis) had MIC values for KAN and AMK above the CC. Conversely, many genotypic wild-type isolates with MIC values higher than CCs were found for several drugs, especially PAS. There are several explanations for this spectrum of results. First, not all mutations confer the same resistance level. The WHO suggested that some mutations confer low, some moderate and some high resistance-levels [5]. Isolates harboring low resistance-level mutations might have MIC values close to the CC. Second, mutation databases are incomplete, especially for the second-line drugs, which might explain why isolates without known resistance-conferring mutations had MIC values higher than the CC. In addition, other drug-resistance mechanisms such as epigenetic mechanisms cannot be identified by genetic analysis [31]. The efflux pump [32] mechanism might fall into this category. Furthermore, we noted that available *in silico* tools were unable to detect certain heteroresistance in *rrs* and *gryA* and gave a false genotypically susceptible result compared to our in-house analysis pipeline for particular drugs. The improvement of the drug-resistance mutation databases, web-based analysis tools and/or use of deep-sequencing techniques [33] might enhance the sensitivity for identification of heteroresistance. Readjustment of CCs for problematic drugs such as EMB [34] and MXF [24, 26], might also help to overcome these problems.

There are previous reports of mutations in genes associated with low MIC levels for INH (*inhA* promoter: -15 c/t promoter [27]), EMB (*embB*: Gly406Asp and Met306Ile [35]), STR (*gidB* [36]), MXF (*gryA*: Asp94Ala [37]), and RFB (*rpoB*: Asp435Val and Asp435Tyr [26, 28, 29]). However, few of these studies had adequate sample sizes [27, 37]. We used multiple M/XDR-TB isolates to test for an association between MIC levels and mutations and found a significant association of the *inhA* promoter -15 c/t, *embB* Gly406Asp, *gid* mutations, *gryA* Ala90Val and *rpoB* Asp435Phe/Tyr/Val with low MIC levels spanning the CCs for INH, EMB, STR, MXF and RFB, respectively. However, the low number of resistance-conferring alleles found in our M/XDR-TB isolates limited our ability to investigate other drugs. The WHO database of mutations associated with resistance [5] is still limited in both number of isolates for each mutation and number of drugs. Our findings support the WHO database for known mutations associated with low-level resistance (INH resistance: -15 c/t *inhA* promoter and MXF resistance: *gryA* Ala90Val). In addition, our results suggest additional mutations associated with low vs. high resistance levels for EMB (*embB* Gly406Asp vs. *embB* Met306Ile), STR (*gid* Gly73Ala vs. *rpsL* Lys43Arg) and RFB (*rpoB* Asp435Phe/Tyr/Val vs. *rpoB* Ser450Leu). Further studies using a larger number of drug-resistant isolates will provide more insights into the association between particular mutations and MIC values.

Heteroresistance occurs when subpopulations within an isolate vary in their degree of resistance. Heteroresistance commonly arises during intermittent exposure to subtherapeutic drug levels, leading eventually to the generation of fully resistant populations [9]. Better understanding of the relationship between heteroresistance and MIC level should improve the effective treatment of TB [38], but has been the subject of few previous studies [10, 11]. *In-vitro* phenotypic experiments have demonstrated that low frequencies of *Mtb* cells harboring *rpoB* mutations within an isolate are associated with decreased MIC levels for RIF [10]. Only one study has reported a possible association between genotypic heteroresistance (based on WGS data) and MXF phenotypic heteroresistance [11]. In our study, we attempted to analyze the association between genotypic heteroresistance based on the proportion of WGS mapped reads of resistance-conferring SNPs and MIC levels for nine drugs. Only RIF, KAN and AMK seemed to show a positive association between read frequencies of relevant mutations and MIC levels. However, the number of genotypically heteroresistant isolates available in our study was also too low for statistical analysis. Overall, our data do indicate a relationship between frequency of resistance-conferring alleles and MIC values in heteroresistant isolates of *Mtb*. This further suggests the considerable applicability of WGS to characterize drug-resistant TB. However, these findings are preliminary, indicating the need for further study with higher sample sizes and systematic analysis.

We found that the WGS method was in good agreement with the MYCOTB system and, for most drugs, in good agreement with the agar proportion test. Although the agar proportion method is still the “gold standard” DST for new drugs for which resistance-conferring mutations are not represented in databases, this method is extremely laborious and time consuming [39]. Similarly, although MIC-based tests can quantify resistance levels, the effort and time required remain obstacles to routine use [39]. The WGS method can shorten the turnaround time, especially when analyzed directly from the samples, and also provides the clustering information needed for epidemiological management [40]. The WGS method provides high-resolution information regarding drug susceptibility and level of resistance. However, a complete database of relevant mutations for each drug and the association of each mutation with resistance level is needed. Our study has contributed part of this information and reinforces the applicability of the WGS method for DST.

Other limitations of our study should be noted. We included a collection of drug-resistant isolates from TB patients in Thailand, including MDR-TB, Pre-XDR-TB and XDR-TB cases. We used these to highlight the effect of drug resistance-conferring mutations on quantitative DSTs for both first-line and second-line anti-TB drugs, except for PZA. PZA is difficult to to use in an agar-based DST because it requires acidity of the culture medium for drug activity [41] and this drug was not included in the MYCOTB MIC plate. Hence, we could not determine the interrelation between phenotypic DST of this drug and likely PZA resistance-conferring mutations which were identified in 26 (43%) isolates. A phylogenetic tree based on whole-genome variants was inferred to ensure that potentially clonal strains did not affect the association analysis. Although there were two small clusters (each including two isolates) of genetically identical *Mtb* isolates among our samples, only one pair of isolates shared the same drug resistance pattern. Hence, the association results were not confounded by the presence of clonal strains. The diversity of resistance-conferring mutations is generally lower in MDR-TB isolates than in mono- or poly-resistant isolates [42, 43]. Most of our isolates were MDR-TB, Pre-XDR-TB and XDR-TB, which could affect the mutation frequencies and sensitivity comparison between DST methods. The database from TB-Profiler includes some mutations for which there is only a low level of confidence, based on current knowledge, that they are actually associated with resistance. Examples of these are *ethyA* associated with ethionamide resistance and *eis* promoter -8 c/a associated with kanamycin resistance). Low-confidence mutations might affect the ability of the WGS method to detect DR and heteroresistance.

## Conclusions

We compared the agreement between phenotypic (agar proportion method and MIC tests using MYCOTB) and genotypic DSTs (WGS) and highlighted problematic drugs, especially ethambutol and ethionamide, that can yield different results according to the DST method used. Additional information was provided regarding mutations associated with low vs. high resistance levels against INH (-15 c/t *inhA* promoter vs. *katG* Ser315Thr), EMB (*embB* Gly406Asp vs. *embB* Met306Ile), STR (*gid* Gly73Ala vs. *rpsL* Lys43Arg), MXF (*gyrA* Ala90Val vs. *gyrA* Asp94Gly) and RFB (*rpoB* Asp435Phe/Tyr/Val vs. *rpoB* Ser450Leu), but further evaluation with a larger sample size is required. A possible association between genotypic heteroresistance and MIC level was also suggested. These results emphasize the high applicability of WGS for TB diagnosis including determination of drug resistance, mutated allele association with MIC and heteroresistance associated with MIC.

## Supporting information

S1 Fig Phylogenetic analysis of 60 *Mycobacterium tuberculosis* isolates. These isolates fell into lineages 1, 2 and 4. The phylogenetic tree was inferred using the maximum likelihood method with general time reversible and gamma distribution model using 7,880 high-confidence SNPs relative to the H37Rv reference genome. The bootstrap consensus tree was inferred from 1,000 replicates. Blue circles refer to bootstrap values and the size of each circle is proportional to its value (most of the bootstrap values are 100). Two small clusters of genetically identical *M*. *tuberculosis* are indicated in grey letters. Only one pair of isolates had the same drug-resistance patterns and hence the association results were not confounded by the presence of clonal strains.
(TIF)

S1 Table Critical concentrations (CCs) used in this study for phenotypic DST assays. (DOCX)

S2 Table Frequency and distribution of drug resistance-conferring mutations on MICs. (DOCX)

## References

[1] World Health Organization. Global Tuberculosis Report 2018: WHO; 2018 Geneva, Switzerland.

[2] Canetti G, Fox W, Khomenko A, Mahler HT, Menon NK, Mitchison DA, et al Advances in techniques of testing mycobacterial drug sensitivity, and the use of sensitivity tests in tuberculosis control programmes. Bull World Health Organ. 1969;41(1):21–43. 5309084 PMC2427409

[3] Witney AA, Gould KA, Arnold A, Coleman D, Delgado R, Dhillon J, et al Clinical application of whole-genome sequencing to inform treatment for multidrug-resistant tuberculosis cases. J Clin Microbiol. 2015;53(5):1473–1483. 10.1128/JCM.02993-14 25673793 PMC4400773

[4] Dookie N, Rambaran S, Padayatchi N, Mahomed S, Naidoo K. Evolution of drug resistance in *Mycobacterium tuberculosis*: a review on the molecular determinants of resistance and implications for personalized care. J Antimicrob Chemother. 2018;73(5):1138–1151. 10.1093/jac/dkx506 29360989 PMC5909630

[5] World Health Organization. The use of next-generation sequencing technologies for the detection of mutations associated with drug resistance in *Mycobacterium tuberculosis* complex: technical guide: WHO; 2018 Geneva, Switzerland.

[6] Bottger EC. The ins and outs of *Mycobacterium tuberculosis* drug susceptibility testing. Clin Microbiol Infect. 2011;17(8):1128–1134. 10.1111/j.1469-0691.2011.03551.x 21631641

[7] Cambau E, Viveiros M, Machado D, Raskine L, Ritter C, Tortoli E, et al Revisiting susceptibility testing in MDR-TB by a standardized quantitative phenotypic assessment in a European multicentre study. J Antimicrob Chemother. 2015;70(3):686–696. 10.1093/jac/dku438 25587993

[8] Faksri K, Kaewprasert O, Ong RT, Suriyaphol P, Prammananan T, Teo YY, et al Comparisons of whole-genome sequencing and phenotypic drug susceptibility testing for *Mycobacterium tuberculosis* causing MDR-TB and XDR-TB in Thailand. Int J Antimicrob Agents. 2019;54(2):109–116. 10.1016/j.ijantimicag.2019.04.004 30981926

[9] Muller B, Borrell S, Rose G, Gagneux S. The heterogeneous evolution of multidrug-resistant *Mycobacterium tuberculosis*. Trends Genet. 2013;29(3):160–169. 10.1016/j.tig.2012.11.005 23245857 PMC3594559

[10] Zhang Z, Wang Y, Pang Y, Liu C. Comparison of different drug susceptibility test methods to detect rifampin heteroresistance in *Mycobacterium tuberculosis*. Antimicrob Agents Chemother. 2014;58(9):5632–5635. 10.1128/AAC.02778-14 25022589 PMC4135805

[11] Operario DJ, Koeppel AF, Turner SD, Bao Y, Pholwat S, Banu S, et al Prevalence and extent of heteroresistance by next generation sequencing of multidrug-resistant tuberculosis. PLoS One. 2017;12(5):e0176522 10.1371/journal.pone.0176522 28545050 PMC5436647

[12] Larsen MH, Biermann K, Tandberg S, Hsu T, Jacobs WR Jr. Genetic Manipulation of *Mycobacterium tuberculosis*. Curr Protoc Microbiol. 2007;Chapter 10:Unit 10A 12. 10.1002/9780471729259.mc10a02s6 18770603

[13] Kent PT, GP K. Public Health Mycobacteriology: A Guide for the Level III Laboratory: Department of Health and Human Services, Centers for Disease Control and Prevention; 1985 Atlanta, Georgia.

[14] Hall L, Jude KP, Clark SL, Dionne K, Merson R, Boyer A, et al Evaluation of the Sensititre MycoTB plate for susceptibility testing of the *Mycobacterium tuberculosis* complex against first- and second-line agents. J Clin Microbiol. 2012;50(11):3732–3734. 10.1128/JCM.02048-12 22895034 PMC3486270

[15] Banu S, Rahman SM, Khan MS, Ferdous SS, Ahmed S, Gratz J, et al Discordance across several methods for drug susceptibility testing of drug-resistant *Mycobacterium tuberculosis* isolates in a single laboratory. J Clin Microbiol. 2014;52(1):156–163. 10.1128/JCM.02378-13 24172155 PMC3911413

[16] Andrews S. FastQC: A Quality Control tool for High Throughput Sequence Data. Babraham Bioinformatics, Cambridge, United Kingdom 2010.

[17] McKenna A, Hanna M, Banks E, Sivachenko A, Cibulskis K, Kernytsky A, et al The Genome Analysis Toolkit: a MapReduce framework for analyzing next-generation DNA sequencing data. Genome Res. 2010;20(9):1297–1303. 10.1101/gr.107524.110 20644199 PMC2928508

[18] Li H, Handsaker B, Wysoker A, Fennell T, Ruan J, Homer N, et al The Sequence Alignment/Map format and SAMtools. Bioinformatics. 2009;25(16):2078–2079. 10.1093/bioinformatics/btp352 19505943 PMC2723002

[19] Coll F, McNerney R, Preston MD, Guerra-Assuncao JA, Warry A, Hill-Cawthorne G, et al Rapid determination of anti-tuberculosis drug resistance from whole-genome sequences. Genome Med. 2015;7(1):51 10.1186/s13073-015-0164-0 26019726 PMC4446134

[20] Li H. Aligning sequence reads, clone sequences and assembly contigs with BWA-MEM. arXiv. 2013:1303.3997.

[21] Feuerriegel S, Schleusener V, Beckert P, Kohl TA, Miotto P, Cirillo DM, et al PhyResSE: a Web Tool Delineating *Mycobacterium tuberculosis* Antibiotic Resistance and Lineage from Whole-Genome Sequencing Data. J Clin Microbiol. 2015;53(6):1908–1914. 10.1128/JCM.00025-15 25854485 PMC4432036

[22] Kumar S, Stecher G, Li M, Knyaz C, Tamura K. MEGA X: Molecular Evolutionary Genetics Analysis across Computing Platforms. Mol Biol Evol. 2018;35(6):1547–1549. 10.1093/molbev/msy096 29722887 PMC5967553

[23] Letunic I, Bork P. Interactive Tree Of Life (iTOL) v4: recent updates and new developments. Nucleic Acids Res. 2019;47(W1):W256–W259. 10.1093/nar/gkz239 30931475 PMC6602468

[24] Gygli SM, Keller PM, Ballif M, Blochliger N, Homke R, Reinhard M, et al Whole-Genome Sequencing for Drug Resistance Profile Prediction in *Mycobacterium tuberculosis*. Antimicrob Agents Chemother. 2019;63(4). 10.1128/AAC.02175-18 30718257 PMC6496161

[25] Dover LG, Alahari A, Gratraud P, Gomes JM, Bhowruth V, Reynolds RC, et al EthA, a common activator of thiocarbamide-containing drugs acting on different mycobacterial targets. Antimicrob Agents Chemother. 2007;51(3):1055–1063. 10.1128/AAC.01063-06 17220416 PMC1803108

[26] Heyckendorf J, Andres S, Koser CU, Olaru ID, Schon T, Sturegard E, et al What Is Resistance? Impact of Phenotypic versus Molecular Drug Resistance Testing on Therapy for Multi- and Extensively Drug-Resistant Tuberculosis. Antimicrob Agents Chemother. 2018;62(2). 10.1128/AAC.01550-17 29133554 PMC5786814

[27] Lempens P, Meehan CJ, Vandelannoote K, Fissette K, de Rijk P, Van Deun A, et al Isoniazid resistance levels of *Mycobacterium tuberculosis* can largely be predicted by high-confidence resistance-conferring mutations. Sci Rep. 2018;8(1):3246 10.1038/s41598-018-21378-x 29459669 PMC5818527

[28] Jamieson FB, Guthrie JL, Neemuchwala A, Lastovetska O, Melano RG, Mehaffy C. Profiling of rpoB mutations and MICs for rifampin and rifabutin in *Mycobacterium tuberculosis*. J Clin Microbiol. 2014;52(6):2157–2162. 10.1128/JCM.00691-14 24740074 PMC4042728

[29] Farhat MR, Sixsmith J, Calderon R, Hicks ND, Fortune SM, Murray M. Rifampicin and rifabutin resistance in 1003 *Mycobacterium tuberculosis* clinical isolates. J Antimicrob Chemother. 2019;74(6):1477–1483. 10.1093/jac/dkz048 30793747 PMC6524487

[30] Miotto P, Tessema B, Tagliani E, Chindelevitch L, Starks AM, Emerson C, et al A standardised method for interpreting the association between mutations and phenotypic drug resistance in *Mycobacterium tuberculosis*. Eur Respir J. 2017;50(6). 10.1183/13993003.01354-2017 29284687 PMC5898944

[31] Freihofer P, Akbergenov R, Teo Y, Juskeviciene R, Andersson DI, Bottger EC. Nonmutational compensation of the fitness cost of antibiotic resistance in mycobacteria by overexpression of tlyA rRNA methylase. RNA. 2016;22(12):1836–1843. 10.1261/rna.057257.116 27698071 PMC5113204

[32] Pule CM, Sampson SL, Warren RM, Black PA, van Helden PD, Victor TC, et al Efflux pump inhibitors: targeting mycobacterial efflux systems to enhance TB therapy. J Antimicrob Chemother. 2016;71(1):17–26. 10.1093/jac/dkv316 26472768

[33] Rigouts L, Miotto P, Schats M, Lempens P, Cabibbe AM, Galbiati S, et al Fluoroquinolone heteroresistance in *Mycobacterium tuberculosis*: detection by genotypic and phenotypic assays in experimentally mixed populations. Sci Rep. 2019;9(1):11760 10.1038/s41598-019-48289-9 31409849 PMC6692311

[34] World Health Organization. Technical report on critical concentrations for drug susceptibility testing of medicines used in the treatment of drug-resistant tuberculosis: WHO; 2018 Geneva, Switzerland.

[35] Ruesen C, Riza AL, Florescu A, Chaidir L, Editoiu C, Aalders N, et al Linking minimum inhibitory concentrations to whole genome sequence-predicted drug resistance in *Mycobacterium tuberculosis* strains from Romania. Sci Rep. 2018;8(1):9676 10.1038/s41598-018-27962-5 29946139 PMC6018741

[36] Wong SY, Lee JS, Kwak HK, Via LE, Boshoff HI, Barry CE 3rd. Mutations in gidB confer low-level streptomycin resistance in *Mycobacterium tuberculosis*. Antimicrob Agents Chemother. 2011;55(6):2515–2522. 10.1128/AAC.01814-10 21444711 PMC3101441

[37] Chien JY, Chiu WY, Chien ST, Chiang CJ, Yu CJ, Hsueh PR. Mutations in gyrA and gyrB among Fluoroquinolone- and Multidrug-Resistant *Mycobacterium tuberculosis* Isolates. Antimicrob Agents Chemother. 2016;60(4):2090–2096. 10.1128/AAC.01049-15 26787695 PMC4808166

[38] Ellington MJ, Ekelund O, Aarestrup FM, Canton R, Doumith M, Giske C, et al The role of whole genome sequencing in antimicrobial susceptibility testing of bacteria: report from the EUCAST Subcommittee. Clin Microbiol Infect. 2017;23(1):2–22. 10.1016/j.cmi.2016.11.012 27890457

[39] Schon T, Miotto P, Koser CU, Viveiros M, Bottger E, Cambau E. *Mycobacterium tuberculosis* drug-resistance testing: challenges, recent developments and perspectives. Clin Microbiol Infect. 2017;23(3):154–160. 10.1016/j.cmi.2016.10.022 27810467

[40] Pankhurst LJ, Del Ojo Elias C, Votintseva AA, Walker TM, Cole K, Davies J, et al Rapid, comprehensive, and affordable mycobacterial diagnosis with whole-genome sequencing: a prospective study. Lancet Respir Med. 2016;4(1):49–58. 10.1016/S2213-2600(15)00466-X 26669893 PMC4698465

[41] Jonmalung J, Prammananan T, Leechawengwongs M, Chaiprasert A. Surveillance of pyrazinamide susceptibility among multidrug-resistant *Mycobacterium tuberculosis* isolates from Siriraj Hospital, Thailand. BMC Microbiol. 2010;10:223 10.1186/1471-2180-10-223 20727143 PMC2942842

[42] Walker TM, Kohl TA, Omar SV, Hedge J, Del Ojo Elias C, Bradley P, et al Whole-genome sequencing for prediction of *Mycobacterium tuberculosis* drug susceptibility and resistance: a retrospective cohort study. Lancet Infect Dis. 2015;15(10):1193–1202. 10.1016/S1473-3099(15)00062-6 26116186 PMC4579482

[43] Jajou R, van der Laan T, de Zwaan R, Kamst M, Mulder A, de Neeling A, et al WGS more accurately predicts susceptibility of *Mycobacterium tuberculosis* to first-line drugs than phenotypic testing. J Antimicrob Chemother. 2019;74(9):2605–2616. 10.1093/jac/dkz215 31119271

