# Supplementary figures and images for: Whole-genome sequence analysis and comparisons between drug-resistance mutations and minimum inhibitory concentrations of Mycobacterium tuberculosis isolates causing M/XDR-TB
Source: PLoS One. 2020 Dec 31;15(12):e0244829. doi: 10.1371/journal.pone.0244829 (PMC7775048; doi:10.1371/journal.pone.0244829)

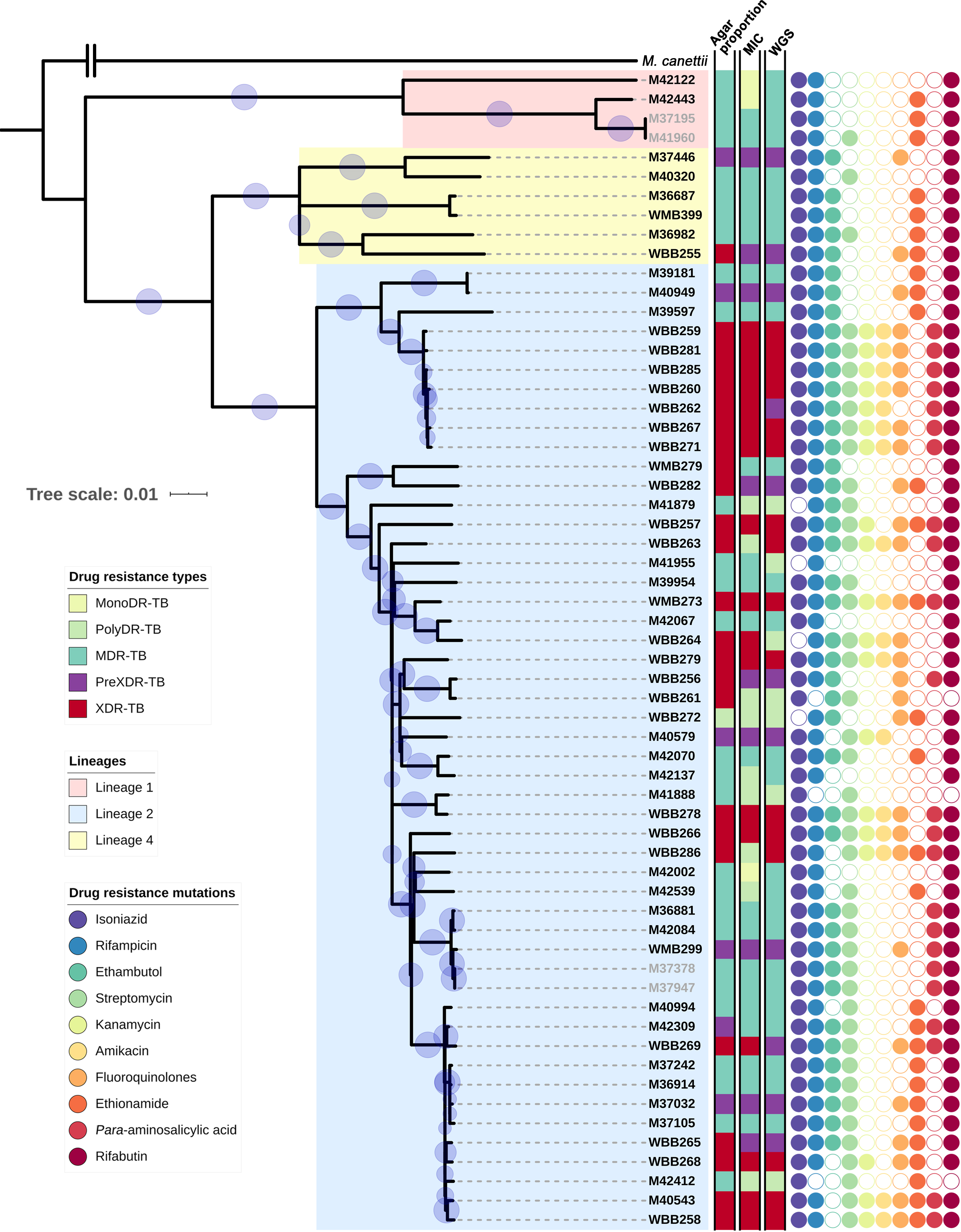

Supplement: S1 Fig — These isolates fell into lineages 1, 2 and 4. The phylogenetic tree was inferred using the maximum likelihood method with general time reversible and gamma distribution model using 7,880 high-confidence SNPs relative to the H37Rv reference genome. The bootstrap consensus tree was inferred from 1,000 replicates. Blue circles refer to bootstrap values and the size of each circle is proportional to its value (most of the bootstrap values are 100). Two small clusters of genetically identical M. tuberculosis are indicated in grey letters. Only one pair of isolates had the same drug-resistance patterns and hence the association results were not confounded by the presence of clonal strains. (TIF) [file pone.0244829.s001.tif]
