# Supplementary material for: Whole-genome sequence analysis and comparisons between drug-resistance mutations and minimum inhibitory concentrations of Mycobacterium tuberculosis isolates causing M/XDR-TB
Source: PLoS One. 2020 Dec 31;15(12):e0244829. doi: 10.1371/journal.pone.0244829 (PMC7775048; doi:10.1371/journal.pone.0244829)
Supplement: S1 Table — (DOCX) [file pone.0244829.s002.docx]

**S1 Table. Critical concentrations used in this study for phenotypic DST assays.**

| **Drug** | **Agar proportion (µg/ml)** | **MYCOTB (µg/ml)** |
| --- | --- | --- |
| **Isoniazid** | 0.2 | 0.25^b^ |
| **Rifampicin** | 1 | 1 |
| **Ethambutol** | 5 | 4^b^ |
| **Streptomycin** | 2 | 2^b^ |
| **Kanamycin** | 6^a^ | 5^b^ |
| **Amikacin** | 6^a^ | 4^b^ |
| **Ofloxacin** | 2 | 2 |
| **Moxifloxacin** | 2 | 1^b^ |
| **Ethionamide** | 5 | 5 |
| ***Para*-aminosalicylic acid** | 2 | 1^b^ |
| **Rifabutin** | - | 0.5 |
| **D-cycloserine** | - | 32 |

Critical concentrations (CCs) that were different from those previously recommended by WHO, following

^a^ Updated recommendations of CCs from WHO [[1](#_ENREF_1), [2](#_ENREF_2)],

^b^ Recommendations of CCs accompanying MYCOTB kit [[3](#_ENREF_3), [4](#_ENREF_4)].

**References**

1. World Health Organization. Companion handbook to the WHO guidelines for the programmatic management of drug-resistant tuberculosis: WHO; 2014. Geneva, Switzerland.

2. World Health Organization. Technical report on critical concentrations for drug susceptibility testing of medicines used in the treatment of drug-resistant tuberculosis: WHO; 2018. Geneva, Switzerland.

3. Hall L, Jude KP, Clark SL, Dionne K, Merson R, Boyer A, et al. Evaluation of the Sensititre MycoTB plate for susceptibility testing of the *Mycobacterium tuberculosis* complex against first- and second-line agents. J Clin Microbiol. 2012;50(11):3732-3734. doi: 10.1128/JCM.02048-12 PMID: 22895034

4. Banu S, Rahman SM, Khan MS, Ferdous SS, Ahmed S, Gratz J, et al. Discordance across several methods for drug susceptibility testing of drug-resistant *Mycobacterium tuberculosis* isolates in a single laboratory. J Clin Microbiol. 2014;52(1):156-163. doi: 10.1128/JCM.02378-13 PMID: 24172155
