# Supplementary material for: Whole-genome sequence analysis and comparisons between drug-resistance mutations and minimum inhibitory concentrations of Mycobacterium tuberculosis isolates causing M/XDR-TB
Source: PLoS One. 2020 Dec 31;15(12):e0244829. doi: 10.1371/journal.pone.0244829 (PMC7775048; doi:10.1371/journal.pone.0244829)
Supplement: S2 Table — (DOCX) [file pone.0244829.s003.docx]

**S2 Table. Frequency and MIC-distribution of isolates with drug resistance-conferring mutations.**

| **Drug** | **Mutations^a^** | **MIC (µg/ml) distribution for relevant isolate** | | | | | | | | | **No. of isolates** | **%** |
| --- | --- | --- | --- | --- | --- | --- | --- | --- | --- | --- | --- | --- |
| **Isoniazid** |  | **≤0.03** | **0.06** | **0.12** | **0.25** | **0.5** | **1** | **2** | **4** | **>4** |  |  |
| (CC = 0.25 µg/ml) | No INH mutations |  |  | 1 | 1 | 1 |  | 1 |  |  | 4 | 7 |
|  | *katG* Ser315Thr |  |  |  |  |  | 12 | 21 | 9 | 1 | 43 | 72 |
|  | *katG* Ser315Thr, *katG* Ala424Gly |  |  |  |  |  |  |  | 1 |  | 1 | 2 |
|  | *katG* Ser315Asn |  |  |  |  |  | 1 |  |  |  | 1 | 2 |
|  | *inhA* -15 c/t |  |  | 1 | 1 | 2 | 1 |  |  |  | 5 | 8 |
|  | *inhA* -15 c/t, *inhA* Ser94Ala |  |  |  |  |  |  |  |  | 1 | 1 | 2 |
|  | *inhA* -15 c/t, *katG* Met257Ile |  |  |  |  |  | 1 |  |  |  | 1 | 2 |
|  | *inhA* -8 t/c, *katG* Ser315Thr |  |  |  |  |  |  |  | 1 | 1 | 2 | 3 |
|  | *inhA* Ser94Ala |  |  |  |  | 2 |  |  |  |  | 2 | 3 |
| **Rifampicin** |  | **≤0.12** | **0.25** | **0.5** | **1** | **2** | **4** | **8** | **16** | **>16** |  |  |
| (CC = 1 µg/ml) | No RIF mutations | 1 | 1 |  | 1 |  |  |  |  |  | 3 | 5 |
|  | *rpoB* Ser450Leu |  |  |  |  |  |  | 1 |  | 35 | 36 | 60 |
|  | *rpoB* His445Arg |  |  |  |  |  |  |  |  | 2 | 2 | 3 |
|  | *rpoB* Ser450Leu, *rpoC* Leu527Val |  |  |  |  |  |  |  |  | 1 | 1 | 2 |
|  | *rpoB* His445Leu |  |  |  |  | 1 |  |  |  |  | 1 | 2 |
|  | *rpoB* His445Tyr |  |  |  |  |  |  |  |  | 2 | 2 | 3 |
|  | *rpoB* Asp435Phe |  |  |  |  |  | 1 | 1 |  |  | 2 | 3 |
|  | *rpoB* Asp435Tyr |  |  |  | 1 |  |  |  |  |  | 1 | 2 |
|  | *rpoB* Asp435Val |  |  |  |  |  |  | 1 |  | 1 | 2 | 3 |
|  | *rpoB* Leu452Pro |  | 1 | 1 |  | 1 |  |  |  |  | 3 | 5 |
|  | *rpoB* Ser441Leu |  | 1 |  |  |  |  |  | 1 |  | 2 | 3 |
|  | *rpoB* Ser450Trp |  | 1 |  |  |  |  |  |  |  | 1 | 2 |
|  | *rpoB* Val170Phe |  |  |  |  | 1 |  |  |  |  | 1 | 2 |
|  | *rpoB* 1295_1303del |  |  |  | 1 |  |  |  |  |  | 1 | 2 |
|  | *rpoB* 1295_1303del, *rpoB* Ser450Leu |  |  |  |  |  | 1 |  |  |  | 1 | 2 |
|  | *rpoB* Leu430Arg, *rpoB* Asp435Tyr |  |  |  |  |  |  |  |  | 1 | 1 | 2 |
| **Ethambutol** |  | **≤0.5** | **1** | **2** | **4** | **8** | **16** | **32** | **>32** |  |  |  |
| (CC = 4 µg/ml) | No EMB mutations | 1 | 7 | 7 | 3 |  |  |  |  |  | 18 | 30 |
|  | *embB* Met306Ile |  |  | 1 | 6 | 6 | 3 |  |  |  | 16 | 27 |
|  | *embB* Gly406Asp |  |  | 1 | 5 |  |  |  |  |  | 6 | 10 |
|  | *embB* Met306Val |  |  |  | 1 | 2 | 1 |  |  |  | 4 | 7 |
|  | *embB* Tyr319Ser |  |  |  | 1 | 3 |  |  |  |  | 4 | 7 |
|  | *embB* Gln497Arg |  |  |  | 1 | 1 |  |  |  |  | 2 | 3 |
|  | *embB* Asp328Tyr |  |  |  |  | 1 |  |  |  |  | 1 | 2 |
|  | *embB* Asp354Ala |  |  | 1 |  |  |  |  |  |  | 1 | 2 |
|  | *embB* Gly406Cys |  |  |  |  | 1 |  |  |  |  | 1 | 2 |
|  | *embB* Met306Leu |  |  |  | 1 |  |  |  |  |  | 1 | 2 |
|  | *embB* Asp1024Asn, *embB* Met306Ile |  |  |  |  | 2 |  |  |  |  | 2 | 3 |
|  | *embB* Asp1024Asn, *embB* Gly406Ser |  |  |  | 1 |  |  |  |  |  | 1 | 2 |
|  | *embA* -12 c/t |  |  | 2 |  |  |  |  |  |  | 2 | 3 |
|  | *embB* Asp328Tyr, *embA* -16 c/t |  |  |  |  | 1 |  |  |  |  | 1 | 2 |
| **Streptomycin** |  | **≤0.25** | **0.5** | **1** | **2** | **4** | **8** | **16** | **32** | **>32** |  |  |
| (CC = 2 µg/ml) | No STR mutations | 10 | 5 |  | 1 | 1 |  |  |  |  | 17 | 28 |
|  | *rpsL* Lys43Arg |  |  |  |  |  |  |  |  | 28 | 28 | 47 |
|  | *rpsL* Lys88Arg |  |  |  |  | 2 |  | 2 | **1** | 1 | 6 | 10 |
|  | *gid* Gly73Ala |  |  | 1 | 1 | 3 | **1** |  |  |  | 6 | 10 |
|  | *gid* 115_115del |  |  |  | 1 |  |  |  |  |  | 1 | 2 |
|  | *gid*_Chromosome:g.4407954_4408172del |  | 1 |  |  |  |  |  |  |  | 1 | 2 |
|  | *rpsL* Lys88Arg, *gid* Gly73Ala |  |  |  |  | 1 |  |  |  |  | 1 | 2 |
| **Kanamycin** |  | **≤0.6** | **1.2** | **2.5** | **5** | **10** | **20** | **40** | **>40** |  |  |  |
| (CC = 5 µg/ml) | No KAN mutations | 11 | 23 | 6 |  |  |  |  | 1 |  | 41 | 68 |
|  | *rrs* A1401G |  |  |  |  |  |  |  | 17 |  | 17 | 28 |
|  | *eis* -14 c/t |  |  |  |  | 1 |  |  |  |  | 1 | 2 |
|  | *eis* -8 c/a |  |  |  |  | 1 |  |  |  |  | 1 | 2 |
| **Amikacin** |  | **≤0.12** | **0.25** | **0.5** | **1** | **2** | **4** | **8** | **16** | **>16** |  |  |
| (CC = 4 µg/ml) | No AMK mutations | 4 | 26 | **9** | 3 |  |  |  |  | 1 | 43 | 72 |
|  | *rrs* A1401G |  |  |  |  |  |  | 2 | **1** | 14 | 17 | 28 |
| **Ofloxacin** |  | **≤0.25** | **0.5** | **1** | **2** | **4** | **8** | **16** | **32** | **>32** |  |  |
| (CC = 2 µg/ml) | No FQ mutations | 1 | 12 | 15 | 3 |  | 1 |  |  |  | 32 | 53 |
|  | *gyrA* Asp94Gly |  |  |  |  | 1 | 7 | 6 | 1 |  | 15 | 25 |
|  | *gyrA* Ala90Val |  |  |  |  | 3 | 2 |  | 1 |  | 6 | 10 |
|  | *gyrA* Asp94Asn |  |  |  |  |  | 1 | 1 |  |  | 2 | 3 |
|  | *gyrA* Asp94His |  |  |  |  |  | 2 |  |  |  | 2 | 3 |
|  | *gyrA* Asp94Ala |  |  |  |  |  | 1 |  |  |  | 1 | 2 |
|  | *gyrA* Ala90Val, *gyrA* Asp94Tyr |  |  |  |  | 1 |  |  |  |  | 1 | 2 |
|  | *gyrA* Asp94Asn, *gyrA* Ala90Val |  |  |  |  | 1 |  |  |  |  | 1 | 2 |
| **Moxifloxacin** |  | **≤0.06** | **0.12** | **0.25** | **0.5** | **1** | **2** | **4** | **8** | **>8** |  |  |
| (CC = 1 µg/ml) | No FQ mutations | 2 | 9 | 13 | 7 |  | 1 |  |  |  | 32 | 53 |
|  | *gyrA* Asp94Gly |  |  |  |  |  | 4 | **11** |  |  | 15 | 25 |
|  | *gyrA* Ala90Val |  |  |  |  | 3 | 2 | **1** |  |  | 6 | 10 |
|  | *gyrA* Asp94Asn |  |  |  |  |  |  | **1** | 1 |  | 2 | 3 |
|  | *gyrA* Asp94His |  |  |  |  |  | 1 | **1** |  |  | 2 | 3 |
|  | *gyrA* Asp94Ala |  |  |  |  |  | 1 |  |  |  | 1 | 2 |
|  | *gyrA* Ala90Val, *gyrA* Asp94Tyr |  |  |  |  | 1 |  |  |  |  | 1 | 2 |
|  | *gyrA* Asp94Asn, *gyrA* Ala90Val |  |  |  |  |  | 1 |  |  |  | 1 | 2 |
| **Ethionamide** |  | **≤0.3** | **0.6** | **1.2** | **2.5** | **5** | **10** | **20** | **40** | **>40** |  |  |
| (CC = 5 µg/ml) | No ETO mutations | 1 | 11 | 16 | 3 |  |  |  |  |  | 31 | 52 |
|  | *ethA* 639_640del |  | 1 | 4 | 3 | 2 | 1 |  |  |  | 11 | 18 |
|  | *ethA* 704_707del |  |  |  |  | 2 |  |  |  |  | 2 | 3 |
|  | *inhA* Ser94Ala |  |  |  |  |  |  |  |  | 2 | 2 | 3 |
|  | *inhA* -15 c/t |  |  | 1 | 1 | 2 | 2 |  |  |  | 6 | 10 |
|  | *ethA* 32_33insG |  |  |  | 1 |  |  |  |  |  | 1 | 2 |
|  | *ethA* 456_456del |  |  |  | 1 |  |  |  |  |  | 1 | 2 |
|  | *ethA* 489_531del, *ethA*_Chromosome:g.43269 |  |  |  | 1 |  |  |  |  |  | 1 | 2 |
|  | *ethA* 551_552insG |  |  | 1 |  |  |  |  |  |  | 1 | 2 |
|  | *ethA* Thr232Ala |  | 1 |  |  |  |  |  |  |  | 1 | 2 |
|  | *inhA* -15 c/t, *inhA* Ser94Ala |  |  |  |  |  | 1 |  |  |  | 1 | 2 |
|  | *inhA* -8 t/c, *ethA* 1047_1047del |  |  |  |  | 1 |  |  |  |  | 1 | 2 |
|  | *inhA* -8 t/c, *ethA* 639_640del |  |  |  | 1 |  |  |  |  |  | 1 | 2 |
| ***Para*-aminosalicylic acid** |  | **≤0.5** | **1** | **2** | **4** | **8** | **16** | **32** | **64** | **>64** |  |  |
| (CC = 1 µg/ml) | No PAS mutations | 25 | 7 | **2** |  |  |  | 2 |  | 1 | 37 | 62 |
|  | *folC* Glu40Gly |  |  | **1** | 4 |  | 1 | 2 |  | 1 | 9 | 15 |
|  | *folC* Ser150Gly |  |  |  | 1 | 1 | 2 |  |  | 2 | 6 | 10 |
|  | *folC* Glu153Ala |  |  |  |  |  |  |  | 1 |  | 1 | 2 |
|  | *folC* Glu153Gly |  |  |  |  | 1 |  |  |  |  | 1 | 2 |
|  | *folC* Ile43Thr |  |  |  | 1 |  |  |  |  |  | 1 | 2 |
|  | *thyX* -16 c/t |  | 2 | **1** |  |  |  |  |  |  | 3 | 5 |
|  | *thyA* Thr22Ala |  | 1 |  |  |  |  |  |  |  | 1 | 2 |
|  | *thyA*_Chromosome:g.3073680_3074470del, *thyX* -16 c/t |  |  | **1** |  |  |  |  |  |  | 1 | 2 |
| **Rifabutin** |  | **≤0.12** | **0.25** | **0.5** | **1** | **2** | **4** | **8** | **16** | **>16** |  |  |
| (CC = 0.5 µg/ml) | No RIF mutations | 1 |  | **1** |  | 1 |  |  |  |  | 3 | 5 |
|  | *rpoB* Ser450Leu |  | 1 | **6** | 10 | 5 | 8 | 5 | 1 |  | 36 | 60 |
|  | *rpoB* His445Arg |  |  |  | 1 | 1 |  |  |  |  | 2 | 3 |
|  | *rpoB* Ser450Leu, *rpoC* Leu527Val |  |  |  | 1 |  |  |  |  |  | 1 | 2 |
|  | *rpoB* His445Leu | 1 |  |  |  |  |  |  |  |  | 1 | 2 |
|  | *rpoB* His445Tyr |  |  |  | 1 |  | 1 |  |  |  | 2 | 3 |
|  | *rpoB* Asp435Phe |  | 1 |  | 1 |  |  |  |  |  | 2 | 3 |
|  | *rpoB* Asp435Tyr | 1 |  |  |  |  |  |  |  |  | 1 | 2 |
|  | *rpoB* Asp435Val | 1 | 1 |  |  |  |  |  |  |  | 2 | 3 |
|  | *rpoB* Leu452Pro | 2 |  |  |  | 1 |  |  |  |  | 3 | 5 |
|  | *rpoB* Ser441Leu | 2 |  |  |  |  |  |  |  |  | 2 | 3 |
|  | *rpoB* Ser450Trp |  |  |  |  |  | 1 |  |  |  | 1 | 2 |
|  | *rpoB* Val170Phe |  |  |  |  |  | 1 |  |  |  | 1 | 2 |
|  | *rpoB* 1295_1303del | 1 |  |  |  |  |  |  |  |  | 1 | 2 |
|  | *rpoB* 1295_1303del, *rpoB* Ser450Leu |  | 1 |  |  |  |  |  |  |  | 1 | 2 |
|  | *rpoB* Leu430Arg, *rpoB* Asp435Tyr |  |  |  |  | 1 |  |  |  |  | 1 | 2 |

CC, critical concentration; INH, isoniazid; RIF, rifampicin; EMB, ethambutol; STR, streptomycin; KAN, kanamycin; AMK amikacin; FQ, fluoroquinolones; OFX, ofloxacin; MXF, moxifloxacin; ETO, ethionamide; PAS, *para*-aminosalicylic acid; RFB, rifabutin.

**^a^** Drug resistance-conferring mutations used in our study were based on the most recent database from TB-Profiler (https://github.com/jodyphelan/TBProfiler/blob/master/db/tbdb.dr.json).
